# Reports on the Hospitals of the United Kingdom

**Published:** 1912-11-16

**Authors:** Henry Burdett


### REPORTS ON THE

### Hospitals of the United Kingdom.

By SIR HENRY BURDETT, K.C.B., K.C.V.O.

### LXVIII.—Royal Victoria Infirmary, Newcastle-on-Tyne.

This hospital occupies a commanding position on the Moor. The elevations, though unpretentious, are attractive on the whole, and some are distinctly good. The hospital is constructed on the pavilion plan, having one communicating corridor running the whole length of the buildings with the ward blocks and their appurtenances situated at either side of it. There are six main ward pavilions and four smaller pavilions with the administration block in the centre, and the nurses' home at the extreme west of the communicating corridor. We propose to publish a plan and details of the buildings in due course. One general criticism called for is the fact that, although this hospital was designed and erected so recently as 1906, the plan shows a surprising absence of attention to many details which are essential to the perfecting of a modern up-to-date hospital unit. It was not and is not structurally in accordance with the highest administrative requirements of the present day.

### The Business Side of the Work.

The business side of the management is specialised and in the result exact particulars are available for comparative and economical purposes, so that every important matter is carefully gone into, and all decisions in regard to expenditure and everything which appertains to the outlay or raising of money are thoroughly mastered and kept under the strictest control. The same may be said of the plan pursued in regard to repairs and maintenance, in connection with which a regular staff of handicrafts-men is maintained, and from what we saw of their work we should say they have attained a high degree of competence, to the no small advantage of the hospital and its upkeep. The pharmaceutical department of this institution is full of interest and instruction, and Mr. Sydney Dunstan, F.C.S., M.P.S., the pharmacist, is a gentleman of great practical experience, who does invaluable work in many directions in co-operation with the medical staff. We hope in a future number to give full particulars of the work in this department, and to bring out clearly the saving in cost which has been effected and the admirable guarantees which are invariably forthcoming that every drug and galenical used is of the purest and best.

### The Nursing Department.

The nursing and domestic departments were originally organised by Miss Edith McCall Anderson, R.R.C., the present matron of St. George's Hospital. She did most excellent work, but, like her successor, she was handicapped by the inadequacy of the accommodation originally provided for the nursing staff, a number of whom still occupy cubicles, an arrangement which we regard as most undesirable and unnecessary in the case of a great institution like the Royal Infirmary, which has been opened within the last decade. Difficulties of this kind are, however, regarded by the working staff at Newcastle as matters which it is their duty to minimise as much as possible, if they cannot be entirely overcome, and the results secured, first by Miss McCall Anderson and subsequently by her able successor, Miss Lucy Wamsley, reflect the greatest credit upon both of these able administrators. We were much struck by the circumstance that the majority at any rate of the sisters in charge of wards have been trained at Newcastle. It says much for the system pursued, and those responsible for the teaching, that the most careful inspection of the smallest details of ward management demonstrated how thorough that teaching is, and how fully those who gain their certificates have equipped themselves for the efficient discharge of this most difficult office. Everything was in the most perfect order, and there was a smartness and keen desire to secure efficiency everywhere throughout this institution which filled us with admiration. It may be interesting to mention that since August 1911 the term of training has been extended to four years, though a certificate is granted at the end of the third year under a four years' agreement. The payments made to the staff are for each year of training £10, £14, £20 and £26 respectively. In the fourth year the certificated nurses are given the title of staff nurses and rank accordingly.

Another addition which we should like to see in connection with the nursing school and home of the Newcastle Infirmary is a preliminary training school, where the raw material may be thoroughly tested and sifted before probationers are selected and admitted to the infirmary for training. We look forward to the time, too, when Newcastle will have a large and increasing private nursing staff available for its citizens under the management of the Royal Infirmary, which will be served by nurses trained within its walls.

### Ward Furniture and Patients' Rights.

In regard to the furniture, we noticed some ward tables where the drawers and cupboards go right through. Details of these, with illustrations, will be found in The Hospital for October 7, 1911, page 20. It is part of Miss Wamsley's plan to make the comfort of the patients the first consideration, and she has no belief in a recent proposal to deprive the patients of ward lockers and to combine them in one piece of furniture situated in the centre of the ward away from the reach and use of the patients who may be confined to bed. We propose to go into this question pretty fully, and here we may say that, on the whole, having regard to the fact that the voluntary hospital system owes its popularity and importance largely to the fact that, unlike that of the State hospital, the patients, their comfort and interests, are put first, we are inclined to think that on the whole Miss Wamsley's view is the right one and should largely prevail in British hospitals everywhere. Another feature of the wards is the oak chairs, there being one such chair by each bedstead. These chairs form a most attractive feature and one which we believe to be unique or almost unique at the present time. The colours selected for the walls and generally throughout the wards and corridors are excellent.

### The Mortuary.

One good feature of this institution is the excellent accommodation provided for laboratory, clinical, and pharmaceutical work. The same remark applies to the pathological department in all its details. The well-proportioned and spacious post-mortem room, however, would be greatly improved and its hygienic condition increased by being provided with an adequate system of ventilation. It is very remarkable, as our inspections are constantly proving, that the adequate ventilation of operation theatres, larders, store-rooms, mortuary and post-mortem rooms, and many other sections of the modern hospital does not seem to have yet entered into the consideration, if the necessity of ventilation is even recognised by a majority, of British architects who are responsible for modern hospital buildings. Will some architect send us particulars and a plan of any hospital or kindred institution which is adequately ventilated, where abundance of good air is always present in the units just named? They do these things better in Germany, where no architect could live if he failed in these important details of his work.

### The Laundry and Linen.

The organisation of the laundry, and the quality of the linen turned out by this establishment, are of the very highest type. We have nowhere found anything better in connection with a hospital, and the whole arrangements reflect the highest credit upon all who may have had to do with the building up of the present excellent system. Everything is done by simplicity of planning and completeness of organisation to make this important department of a great hospital smooth in its working and absolutely efficient throughout. The system of book-keeping, the arrangement and sorting of the linen, the breaking up of the work into handable sections, the duplicating of the more important and responsible members of the staff, and the introduction of laundry pupils and their thorough training are all features which we commend to the study of hospital administrators generally, and especially to those amongst their number who have to do with the domestic department of a hospital.

### Finance.

The ordinary sources of revenue of a voluntary hospital do not produce at Newcastle anything like the amounts received by similar institutions in large towns throughout England. The subscriptions from individuals show a tendency to diminish rather than to increase. They amounted to only £1,361 in 1911. The subscriptions from firms, however, have grown from £2,579 in 1905 to £3,528 in 1911. In the latter year donations and benefactions were less than £1,000. The Hospital Sunday Fund contribution has fallen from £1,200 in 1906 to £750 in 1911. Country collections, £442, show a slight increase, whilst patients' contributions rose from insignificance to £834 in 1910, and stood at £526 in 1911. These figures, with the exception of the last item, which points to the excellence of the business management that prevails at this institution, are not very encouraging for the voluntary system. Newcastle has, however, its own special source of revenue, which has increased by leaps and bounds during the last five years especially. This source is derived from the systematic contributions of workmen, who through Honorary Agents, being representatives of workmen, and of colliery and works officials, collect and forward the contributions from workshops and establishments. The amount so raised in 1911 was £18,850, a sum equal to more than half of the total expenditure. This is a striking instance of the appreciation of the work done by the infirmary amongst the working classes, who are greatly dependent upon it when suffering from illness or accident. It is a remarkable feature of the system adopted for raising this money at Newcastle that the names of the working-men governors occupy nearly twenty-eight pages of the annual report. These governors have the habit of frequently visiting the hospital, especially on Sundays, and we imagine that the crowds of people sometimes to be found in the wards and other parts of the hospital must add greatly to the expense of upkeep. The cost of the extra labour required to maintain every part of the hospital in the perfect state of cleanliness which is one of its most noteworthy features cannot be small. It says much for the good temper and sense of those responsible for the management, and of the working classes too, that a plan under which every contribution of £10 entitles the contributors to the election of a governor should have continued so long unmodified, and with so little friction. It is the rule to fix a certain maximum number of governors to be elected by contributing workshops, and Newcastle is the only example, we believe, in the country where the working classes, by their contributions, have the right to nominate nearly 1,900 governors every year.

There is a Subscription Committee, an active working body, of which Sir Riley Lord is Chairman, which leaves no source of possible revenue unexploited. In addition to the usual sources, this committee through local committees organises house-to-house collections, is responsible largely for the subscriptions in workshops, and keeps its eye upon the organisation of country committees, demonstrations, social clubs and institutes, and every possible source which seems to offer an opportunity to raise a little money for the infirmary. During 1911 finance was satisfactory, seeing that there was an excess of income over expenditure amounting to £836, as against an excess of expenditure over income in the previous year of upwards of £3,000. This satisfactory result is due mainly to the circumstance that legacies produced £6,275 during 1911.

SOUND CONTROL AND GOOD ADMINISTRATION.

By the exercise of much self-denial and ability, the staff have succeeded in making the best of the buildings, and the management at the present time is undoubtedly of the very best. Newcastle people who become connected with the infirmary require the fullest details of the work undertaken by each department. This necessitates the employment of a large staff in connection with the secretariat, and we doubt if there is any hospital in the kingdom which employs so large and well-trained a staff.

We have visited this hospital on two recent occasions and spent a good many hours there. We have had the opportunity of testing in various practical directions the ability and knowledge of the principal members of the staff and of acquainting ourselves in full detail with the system of administration pursued throughout all departments. We have come to the conclusion that in these days, when it has become the fashion for hospital workers to make excursions to various hospitals and go through them in a body—a plan, by the way, which we deprecate as being least calculated to tend to the increase of useful practical knowledge by individual workers—there are few hospitals where anyone who has a knowledge of hospital affairs and desires to increase it can find more helpful material in fulfilment of these objects than at the Royal Infirmary, Newcastle. Newcastle is a keen business centre. The Infirmary Board have as their principal officer in Mr. Roden Orde, the House Governor and Secretary, an untiring worker and a most capable administrator. He makes it his business to think out and study every hospital question from all points of view; he has sound, clear views in regard to most points of importance, and he exemplifies those views throughout the great institution of which he is the head. It is a great comfort to meet a man in high office who can at once answer a practical question, supply you with every detail proving his thorough knowledge, and demonstrate the care with which every point is thought out and mastered, before any new department or any improvement in an existing department is undertaken. We should like to see more evidence that the middle classes in Newcastle and its neighbourhood took a more liberal and generous interest in the support of the Newcastle Royal Infirmary. There is need, not merely from the infirmary's point of view but from the individual's point of view, for a greater exhibition throughout Newcastle and the district, apart from the working classes, of the awakening of more individual interest in the sick. This should find expression in personal service for the infirmary.

